# Stereological Study of Amygdala Glial Populations in Adolescents and Adults with Autism Spectrum Disorder

**DOI:** 10.1371/journal.pone.0110356

**Published:** 2014-10-17

**Authors:** John T. Morgan, Nicole Barger, David G. Amaral, Cynthia M. Schumann

**Affiliations:** Department of Psychiatry and Behavioral Sciences and the M. I. N. D. Institute, University of California Davis, Sacramento, California, United States of America; Harvard Medical School, United States of America

## Abstract

The amygdala undergoes aberrant development in autism spectrum disorder (ASD). We previously found that there are reduced neuron numbers in the adult postmortem amygdala from individuals with ASD compared to typically developing controls. The current study is a comprehensive stereological examination of four non-neuronal cell populations: microglia, oligodendrocytes, astrocytes, and endothelial cells, in the same brains studied previously. We provide a detailed neuroanatomical protocol for defining each cell type that may be applied to other studies of the amygdala in neurodevelopmental and psychiatric disorders. We then assess whether cell numbers and average volumes differ between ASD and typically developing brains. We hypothesized that a reduction in neuron numbers in ASD might relate to altered immune function and/or aberrant microglial activation, as indicated by increased microglial number and cell body volume. Overall, average non-neuronal cell numbers and volumes did not differ between ASD and typically developing brains. However, there was evident heterogeneity within the ASD cohort. Two of the eight ASD brains displayed strong microglial activation. Contrary to our original hypothesis, there was a trend toward a positive correlation between neuronal and microglial numbers in both ASD and control cases. There were fewer oligodendrocytes in the amygdala of adult individuals with ASD ages 20 and older compared to typically developing controls. This finding may provide a possible sign of altered connectivity or impaired neuronal communication that may change across the lifespan in ASD.

## Introduction

Autism spectrum disorder (ASD) is a neurodevelopmental disorder marked by deficits in social cognition and the presence of restricted and/or repetitive behaviors. It is currently estimated that 1 out of every 68 newborns in the United States will be diagnosed with ASD [1]. The amygdala is a medial temporal lobe structure that modulates social and emotional processing, both of which are disrupted in ASD. It displays numerous functional abnormalities in adolescents and adults with the disorder (e.g., [2]–[6]). In some children with ASD, the amygdala has an aberrant growth trajectory marked by early enlargement followed by normal or even reduced volume by adulthood [7]–[14].

The amygdala has been associated with the neuropathology of ASD from the earliest postmortem studies [8], [15]–[16]. We previously investigated neuron number in the postmortem amygdala from a cohort of adolescent and adult males with ASD and found that there are, on average, fewer neurons in ASD than in typically developing cases [17]. However, the three major glial populations of the brain, microglia, oligodendrocytes, and astrocytes, as well as the endothelial cells, all have not been stereologically examined in either the ASD or typically developing amygdala.

There is evidence that excessive microglial activation may be present in ASD. Studies focusing on frontal and visual cortices and the cerebellum report increased microglial density and aberrant cell morphology in a subset of ASD brains [18]–[20]. Microglia are innate neuroimmune effector cells of the brain, and increase in number and volume when activated (reviews: [21]–[22]). Alterations in cytokine and immune-related gene expression profiles have also been reported in frontal and temporal cortices in ASD, suggesting enhanced microglial activation in the disorder [20], [23]–[24]. Moreover, [^11^C](*R*)-PK11195, a radiotracer that binds to microglia, is elevated across many regions of the brain in ASD [25]. Given that increased microglial activation has been associated with lower neuron numbers in temporal lobe structures in several neurodegenerative conditions [26]–[28], we hypothesized that our finding of reduced neuron numbers in the amygdala in ASD might be related to increased microglial activation in adults with the disorder.

There is also some evidence for aberrant astrocyte activation in the frontal cortex and cerebellum of ASD cases [20]. Astrocytes coordinate innate immune responses and promote environmental homeostasis in the brain. Glial fibrillary acidic protein (GFAP), which is upregulated during astrocyte activation, is increased in multiple brain regions of some ASD cases [20], [24], [29]–[30]. As for oligodendrocytes, there is little evidence of abnormalities in the ASD brain. Oligodendrocytes are the most numerous glial population in the brain and are primarily responsible for myelinating axons. Interestingly, oligodendrocytes have a high vulnerability to environmental perturbations such as microglial activation [31]–[34]. Lastly, endothelial cell abnormalities in ASD have not been investigated. Endothelial cells are the primary cell type that forms the blood vessels innervating the brain. They are primarily responsible for ensuring proper vascularization and the integrity of the blood-brain barrier.

In this study, we stereologically examined the microglia, oligodendrocyte, astrocyte, and endothelial cell populations in the amygdala overall and in five of its major subdivisions. To create a foundation for future cellular studies, we provide a detailed, reliable neuroanatomical protocol for identifying these cell populations. These are the first stereological estimates to be published for the major non-neuronal cellular populations of the typically developing human amygdala. They can also be used as a baseline for future studies of neurodevelopmental and psychiatric disorders that involve the amygdala. We then present a comprehensive stereological study of the non-neuronal cell populations in the amygdala and its nuclei in ASD relative to typically developing brains. This study was carried out using the same cohort of postmortem brains as our previous stereological study of neuron numbers, with the same nuclear definitions. Therefore, an additional goal was to determine whether any non-neuronal alterations, if present, were related to the neuron number alterations previously reported. Because neuron numbers were particularly altered in the lateral nucleus, we focused on it as a region of interest in the study. Finally, due to previous magnetic resonance imaging findings of age-related changes in pathology in the amygdala of ASD brains [7]-[14], we examined whether there were any differences in these cellular populations between adolescent and adult brains.

## Materials and Methods

### Tissue Acquisition and Sectioning

This study utilized postmortem brains from diagnosed ASD (n = 8) and age-matched typically developing (n = 10) cases (Table 1) previously examined in stereological analyses of neuron number [17], [35]. Seven of the eight ASD cases were diagnosed via postmortem administration of the Autism Diagnostic Interview-Revised [36]. The remaining case had extensive medical records available indicating that the donor met full DSM-IV criteria for ASD. All cases were male and had no medical history of other major neurological or psychiatric disorders. Clinical summaries of each ASD case are presented in Information S1.

**Table 1:** Descriptive information for all cases.

Brain bank number	Diagnosis	Age	Hemisphere	PMI	Primary/Secondary Cause of Death
UCD H-2-04	Control	11	Left	30	Cardiac Arrest/Renal Failure
BTB-3830	Control	14	Left	19	Asphyxia
BTB-3831	Control	17	Left	24	Motor Vehicle Accident
BTB-3851	Control	18	Left	20	N/A
BTB-3809	Control	24	Left	19	N/A
BTB-4016	Control	25	Left	20	N/A
BTB-3849	Control	27	Left	21	Arteriosclerotic Cardiovascular Disease
BTB-3706	Control	27	Right	21	Asphyxia
BTB-3966	Control	32	Left	17	N/A
UCD H-19-01	Control	44	Left	26	N/A
BTB-3714	ASD	10	Right	24	Drowning
UCD H-4-99	ASD	15	Right	12	Cardiac Arrest/Renal Failure
AN02736	ASD	15	Right	3	Aspiration
AN11206	ASD	16	Right	48	Undetermined
AN00764	ASD	20	Right	24	Motor Vehicle Accident
UMB-4226	ASD	28	Left	18	Monoxide Poisoning
AN16961	ASD	36	Left	24	Cardiac Arrest/Renal Failure
AN06746	ASD	44	Left	31	Acute Myocardial Infarction

Capsule clinical summaries for ASD cases are provided in Information S1.

ASD, Autism Spectrum Disorder. N/A, not available.

The Institutional Review Board (IRB) Administration of the University of California, Davis reviewed the study design and determined that formal IRB approval of the use of human subjects was not required because the study did not meet US Department of Health and Human Services criteria for a human subjects study as it did not involve living individuals and no private identifiable information was involved. Written informed consent for use of the tissue in postmortem studies was obtained from the next of kin by the brain bank or donation center from which the sample was drawn.

Tissue sectioning for all cases was performed as previously described [17], [35]. Briefly, tissue was removed from the skull and immersed in formalin for a minimum of 2 months. One 4 cm block containing the entire rostrocaudal extent of the amygdala was dissected and placed into cryoprotectant solution (10% glycerol in 0.1 M phosphate buffer for 2 days and 20% glycerol for 5 days) in preparation for freezing. The tissue block was frozen with 2-methyl butane (isopentane) and serially coronally sectioned into six series of 50-µm-thick sections and two series of 100-µm-thick sections. Every other section from one 50 µm series (i.e. one section every 1000 µm) was selected for use in the current immunohistochemistry study as described below.

### Immunohistochemistry

We immunohistochemically stained 50 µm tissue sections for ionized calcium-binding adaptor molecule 1 (Iba1) and counterstained using hematoxylin/eosin (H & E). Additional series from 2 cases (1 ASD case, UMB-4226, and 1 typically developing control case, UCD H-2-04) were stained for glial fibrillary acidic protein (GFAP) and counterstained with H & E to assist in developing our astrocyte nuclei definitions.

Sections were allowed to warm to room temperature, and then washed 3 times via gentle agitation for 1 minute in double distilled water (ddH_2_O) to remove residual cryoprotectant. We then mounted the sections from 0.1 M acetate (C_2_H_3_O_2_) buffer (pH = 6.0) onto 3″×4″ microscope glass slides (Brain Research Laboratories, Newton, MA) and air dried 40 hours prior to immunohistochemistry to maximize section adhesion. Tissue slides were then immersed in 3% hydrogen peroxide (H_2_O_2_) and methanol (MeOH) solution for 30 minutes to block general peroxidase activity and rinsed for 30 seconds in 0.02 M potassium phosphate buffered saline (KPBS) to remove blocking solution. We performed antigen retrieval by heating the tissue slides in 0.1 M citrate (C_6_H_7_O_7_) buffer (pH = 6.0) at 100°C in an uncapped pressure cooker for 50 minutes, followed by a 60 minute cool down period under a 10 pounds per square inch (psi) pressure cap. Tissue slides were washed 3 times via 1 minute immersion in 0.02 M KPBS to remove the citrate buffer.

We then used a wax immunopen to encircle the tissue on the slide to form a well for immunohistochemistry. All tissue slides were stored in a humidity chamber from this step through 3,3′-diaminobenzidine tetrachloride (DAB) development. We incubated tissue slides in a solution of 2% normal goat serum (NGS), 1% bovine serum albumin (BSA), and 0.5% triton x-100 in KPBS at room temperature for 3 hours to permeabilize the tissue and block background staining. Tissue slides were then washed to remove the solution 3 times via 1 minute immersion in 0.02 M KPBS. We then incubated the tissue slides with a rabbit polyclonal primary antibody against Iba1 (1∶1000, Wako USA, Richmond, VA) in all brains. An additional tissue section series from two of the brains was incubated with a primary antibody against GFAP (1∶1000, Dako, Carpenteria, CA). Primary antibodies were delivered in a solution containing 0.5% triton x-100 in 0.02 M KPBS. Tissue slides were incubated in this solution for 40 hours at 4°C then washed 3 times via 1 minute immersion in 0.02 M KPBS to remove the primary antibody solution.

We then incubated the tissue slides with 1∶200 anti-rabbit secondary antibody (Vector Laboratories, Burlingame, CA) in 0.5% triton x-100 in 0.02 M KPBS for 2 hours then washed 3 times via 1 minute immersion in 0.02 M KPBS to remove the solution. The tissue slides were incubated for 1 hour in ABC reagent (ImmunoBioScience, Everett, WA), prepared according to the manufacturer's recommended protocol, with the addition of 0.5% triton x-100, then washed 3 times via 1 minute immersion in 0.02 M KPBS to remove the ABC solution. We then incubated with 1∶200 anti-rabbit secondary antibody (Vector Laboratories, Burlingame, CA) in 0.5% triton x-100 in 0.02 M KPBS for 1 hour and washed 3 times via 1 minute immersion in 0.02 M KPBS to remove the secondary antibody solution. The tissue slides were then incubated for 30 minutes in ABC reagent (ImmunoBioScience, Everett, WA), which was prepared according to the manufacturer's recommended protocol, with the addition of 0.5% triton x-100, and washed 3 times via 1 minute immersion in 50 mM Tris-buffered saline (TBS) in preparation for development. We developed the tissue slides via 10 minute incubation in 0.05% DAB (MP Biomedicals, Solon, OH) in 50 mM TBS, then washed the tissue slides 3 times via 1 minute immersion in 50 mM TBS and air-dried overnight.

We defatted the tissue and removed the wax immunopen wells with 2 immersions in 50% chloroform/50% EtOH that were each 2 hours in length. The tissue sections were rehydrated via a progressive series of 100%/100%/95%/70% EtOH immersions (4 minutes each). Then, we counterstained via full immersion in undiluted H & E (Vector Laboratories) for 5 minutes and dipped briefly in ddH_2_O to remove excess staining. The tissue slides were dehydrated via a progressive series of 50%/70%/95%/95%/100%/100% EtOH immersions (4 minutes each), followed by 3 immersions in 100% xylene washes (4 minutes each). They were then coverslipped (Brain Research Laboratories) with DPX mounting medium (Electron Microscopy Services, Hatfield, PA).

### Anatomical Definitions of the Amygdala and Subdivisions

**Figure 1:**
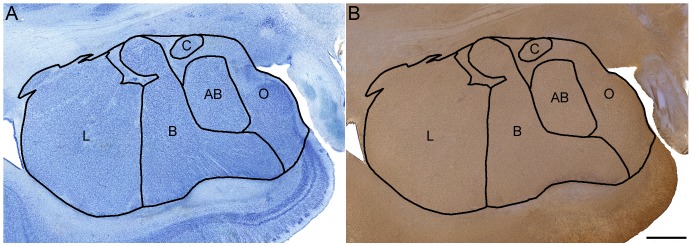
Delineations of the amygdala subregions examined. A. Delineation outlined on a Nissl stained section and B. transferred onto an Iba1- and H & E- stained section and aligned using morphological features, fiber tracts, and cytoarchitectonic boundaries. AB: accessory basal nucleus, B: basal nucleus, C: central nucleus, L: lateral nucleus, O: "other nuclei", a subregion comprising the remaining amygdaloid nuclei, including the anterior cortical nucleus, anterior amygdaloid area, nucleus of the lateral olfactory tract, periamygdaloid cortex, medial nucleus, posterior cortical nucleus, amygdalohippocampal area, and intercalated nuclei. Scale bar: 2 mm.

This study utilized previously published delineations (i.e. contours) for the amygdaloid complex outlined on Nissl stained sections [17], [35] in a series cut in parallel with adjacent Iba1 sections (Figure 1). The delineations defined the whole amygdala as well as divided it into five subregions: (1) lateral nucleus, (2) basal nucleus, (3) accessory basal nucleus, (4) central nucleus, and (5) a region comprising the remaining amygdaloid nuclei not in subdivisions 1-4, including the anterior cortical nucleus, anterior amygdaloid area, nucleus of the lateral olfactory tract, periamygdaloid cortex, medial nucleus, posterior cortical nucleus, amygdalohippocampal area, and intercalated nuclei ("other nuclei"). For each case, the outlined contours were overlaid onto Iba1 immunohistochemically stained sections using gross morphological features (*e.g*., the lateral ventricle) as well as cytoarchitectonic boundaries and fiber tracts that were visible via the H & E counterstain (Figure 1).

### Stereological Measurements

All assessments were conducted on a Nikon Eclipse 80i microscope (Nikon Instruments, Melville, NY) with an Optronics Microfire camera (Optronics, Goleta, CA) using a 100× objective (1.3 NA) (Nikon Instruments) and Köhler illumination via Stereo Investigator 9.0 software (MBF Bioscience, Williston, VT). All stereological quantification was performed with a 1 µm guard zone and a 3 µm counting zone (depth of counting frame). We ensured that the primary Iba1 antibody had completely penetrated the counting zone by examining the bottom 1 µm of the section, beyond the counting zone, and determining that it consistently contained robustly stained Iba1-positive cells. Cells were counted if the topmost point of their H & E counterstained nucleus was in focus within the 3 µm counting zone. The section thickness was measured at every counting site and used as a number-weighted correction factor to correct for variability in tissue shrinkage (Table 2).

**Table 2:** Cell number and microglial somal volume data for the whole amygdala in individual cases, sorted by diagnosis and age.

Case	Diagnosis	Age	Section Thickness	Microglia Number	Microglial Somal Volume	Oligodendrocyte Number	Astrocyte Number	Endothelial Cell Number	Neuron Number
UCD H204	Control	11	6.38	4.67	323.0	19.30	7.20	7.04	10.38
BTB-3830	Control	14	5.39	7.07	261.2	31.60	4.89	4.12	12.90
BTB-3831	Control	17	7.08	8.91	203.0	22.78	10.39	5.55	12.98
BTB-3851	Control	18	8.29	7.59	253.3	30.78	10.10	4.64	11.72
BTB-3809	Control	24	5.99	6.67	269.7	24.29	7.36	4.80	11.71
BTB-4016	Control	25	8.28	7.03	341.2	29.84	10.14	4.49	11.69
BTB-3849	Control	27	7.39	9.63	297.8	33.04	10.62	5.06	14.47
BTB-3706	Control	27	7.12	7.44	210.4	31.46	8.91	3.77	13.71
BTB-3966	Control	32	7.01	7.36	281.8	25.46	11.17	4.79	11.82
UCD H1901	Control	44	7.33	8.26	311.4	37.19	9.19	5.69	10.75
**Mean**	Control	23.9	7.81	7.46	275.3	28.57	9.00	4.99	12.21
BTB-3714	ASD	10	6.57	8.37	250.5	32.36	9.58	6.43	12.39
AN02736	ASD	15	9.62	10.60	375.5	24.79	10.25	4.73	11.30
UCD H499	ASD	15	7.42	8.29	215.3	33.26	9.21	4.86	10.10
AN11206	ASD	16	7.55	6.66	180.9	35.50	10.48	5.58	10.42
AN00764	ASD	20	6.95	10.04	409.0	19.85	9.58	6.48	12.44
UMB-4226	ASD	28	8.27	7.56	286.4	27.57	7.86	4.70	8.63
CAL101	ASD	35	N/A	N/A	N/A	N/A	N/A	N/A	12.30
AN16961	ASD	36	9.31	5.79	241.0	23.34	7.30	4.09	8.87
AN06746	ASD	44	6.80	9.79	238.5	23.24	13.54	7.97	10.19
**Mean**	ASD	23	7.02	8.39	274.6	27.49	9.73	5.60	10.74

All number values are in millions (x10^6^). All section thickness values are in µm. All somal volume values are in µm^3^. N/A, Tissue not available for processing. Neuron number data was originally published in [17], [35]. Equivalent density values are presented in Table S1.

Cells were sampled at a density sufficient to achieve a Gundersen (m = 1) coefficient of error (CE) <.10 in all cases for all cell population estimates in each subdivision of the amygdala (Table 3). The disector used was 60 µm×60 µm in the x-y axis (3600 µm^2^) and 3 µm in the z-axis. The disector x-y spacing (grid size) was set by nucleus: accessory basal nucleus: 715 µm×715 µm, basal nucleus: 950 µm×950 µm, central nucleus: 290 µm×290 µm, lateral nucleus: 1040 µm×1040 µm, other nuclei grouping: 950 µm×950 µm. The average numbers of sampled cells and coefficients of error by nucleus are presented in Table 3.

**Table 3:** Average number of cells sampled and coefficient of error by region, cell type, and diagnosis.

Diagnosis	Cell Type	Information	Lateral Nucleus	Basal Nucleus	Accessory Basal Nucleus	Central Nucleus	Other Nuclei
Control	Microglia	# of cells	310	302	243	302	399
		CE	.06	.06	.07	.07	.05
	Astrocyte	# of cells	423	330	273	321	491
		CE	.05	.06	.06	.06	.04
	Oligodendrocyte	# of cells	1437	1135	786	1068	1320
		CE	.03	.03	.04	.04	.03
	Endothelial Cell	# of cells	236	198	171	217	240
		CE	.07	.07	.08	.08	.07
ASD	Microglia	# of cells	334	314	260	341	423
		CE	.06	.06	.06	.06	.05
	Astrocyte	# of cells	395	350	276	343	489
		CE	.05	.06	.06	.06	.05
	Oligodendrocyte	# of cells	1359	969	701	1166	1197
		CE	.03	.04	.04	.03	.03
	Endothelial Cell	# of cells	256	212	173	250	255
		CE	.07	.07	.08	.07	.07

All glial cell number estimations were conducted via the optical fractionator feature of Stereo Investigator. All glial nucleus and cell body volume estimations were conducted via the isotropic nucleator feature of Stereo Investigator. The H & E stained nucleus volume was estimated for every cell recorded. The Iba1-positive cell body volume was estimated for every microglial cell recorded.

A single rater (JM) collected all data in this study. A reliability assessment was performed in 4 cases for all cell populations, demonstrating>95% intra-rater reliability for each population as determined by intra-class correlation.

### Statistical Analysis

All analyses were conducted using IBM SPSS Statistics 21.0 (IBM Corp., Armonk, NY). Although our sample size was too small to perform a Shapiro-Wilk test for normality, the glial number and volume data did not appear to have a normal distribution. Therefore, we examined the population differences between the ASD and typically developing control cases via non-parametric Mann-Whitney U test. Relationships between cell populations were assessed via non-parametric correlation. To further investigate the presence of developmental effects, we separated the cases into adolescent (age<20) and adult (age 20+) subgroups.

### Image Preparation

**Figure 2:**
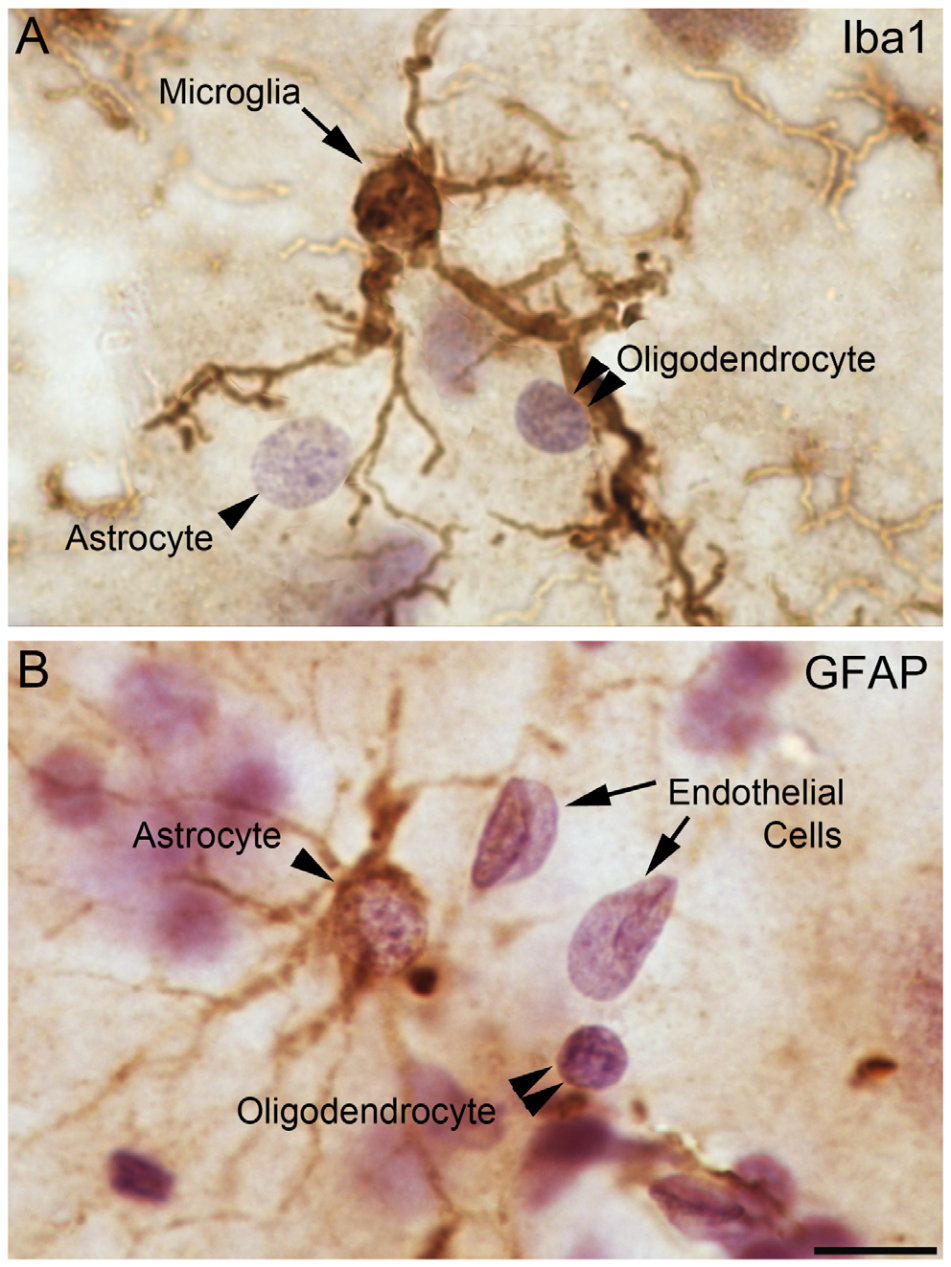
Morphologies of examined cellular populations. Tissue stained brown with A. Iba1-positive primary antibody to identify microglial cells and B. GFAP-positive primary antibody to identify astrocytes. Counterstained purple with H & E. Scale bar: 10 µm.

**Figure 3:**
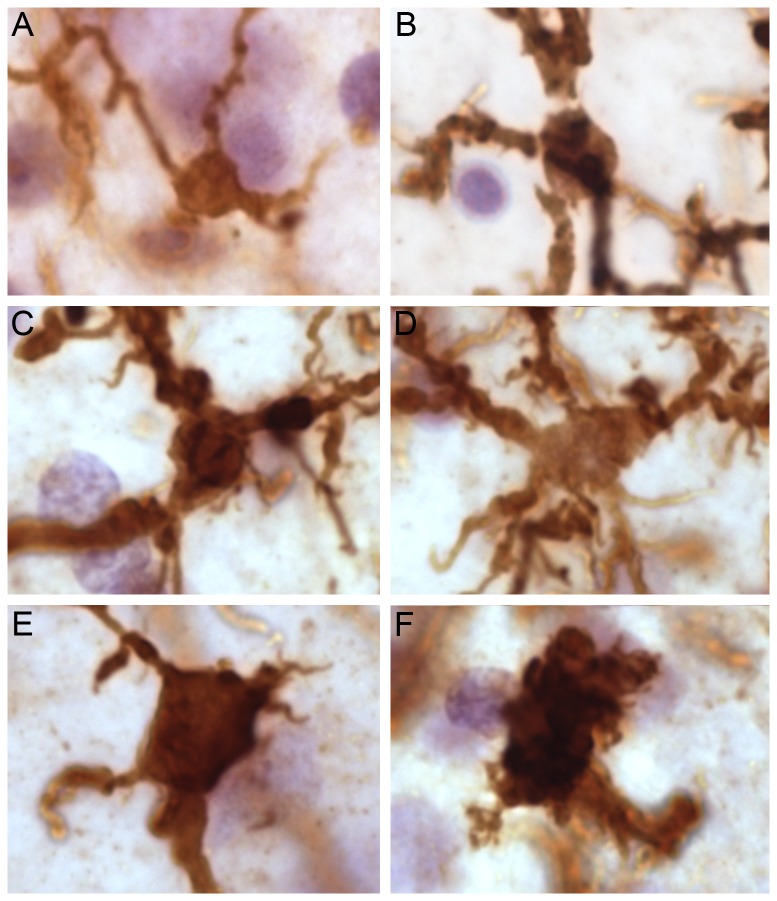
Microglial morphology from resting to very strongly activated in an ASD case that displayed strong microglial activation (AN02736). A. Resting microglial morphology (volume ∼75 µm^3^). B. Slightly activated microglial morphology (volume ∼150 µm^3^). C. Activated microglial morphology (volume ∼275 µm^3^). D. Activated microglial morphology (volume ∼400 µm^3^). E. Strongly activated microglial morphology (volume ∼525 µm^3^) F. Very strongly activated microglial morphology (volume ∼900 µm^3^). Scale bar: 10 µm.

The full microglial arbor was not fully in focus in any single plane of view for image capture. Therefore, we assembled a 3D image montage to visualize all microglial processes in Figure 2. To do this, we acquired serial images from a single section at 1 µm depth intervals. The microglial arbor was reconstructed from the processes that were in focus in each serial image using Adobe Photoshop 11.0.2 (Adobe Systems Incorporated, San Jose, CA USA). This resulted in a flattened, two-dimensional composite of the microglial cell's full arbor within the section. We edited several incidental processes that were not part of the microglial arbor out of the image to make the labels easier to read. Contrast, brightness, and color levels were adjusted to make the image panels more visually consistent in Figures 2, 3, and 6.

## Results

### Glial Cell Definitions

Four cell populations, microglia, oligodendrocytes, astrocytes, and endothelial cells, were identified in the series of tissue sections stained for Iba1 immunoreactivity and counterstained with H & E. The Iba1 immunoreactivity stained the cell bodies of microglial cells specifically, while the H & E stain marked the nuclei of all cell types. We identified microglial cells based on the presence of Iba1-positive staining. The remaining cell types, oligodendrocytes, astrocytes, and endothelial cells, were identified based on their H & E stained nuclear morphology and size, as described below and summarized in Table 4. Several previous studies using Nissl-stained material were used as a reference to develop our protocols for the current study [37]–[39]. Morphological criteria for identifying neurons were previously published [17], [35]. The other cell types are as follows:

**Table 4:** Criteria used to classify glia by nuclear morphology.

Criterion	*Oligodendrocytes*	*Microglia*	*Astrocytes*	*Endothelial Cells*
Size	Small, <6 µm diameter	Variable, 2–6 µm diameter	Large,>5 µm diameter	Large, longest axis>5 µm
Shape	Commonly spherical, with some oval shapes observed.	Highly variable, including spherical, oval, irregularly shaped, and triangular nuclei.	Spherical or oval. Oval shapes are more common than in oligodendrocytes.	Typically folded over, elongated ovals. Rarely very large and oval in large blood vessels.
Staining Intensity	High	Intermediate	Low	Low
Granularity	High with larger granules predominating (usu. 5–15 visible) over smaller ones.	Moderate density of small, light granules with a few larger granules interspersed (usu. 3–6).	High density of smaller, light granules incorporating fewer large granules (usu. 0–4).	Typically exhibit only a moderate density of light granules.
Nucleus Boundary Morphology	Rounded	Rounded or angular (less commonly.)	Rounded	Angular or rounded (less commonly.)
Staining Intensity of Nucleus Boundary	Moderate to dark	Moderate to light	Light	Moderate to dark
Staining of Cytoplasm Around Nucleus	No cytoplasm stained around nucleus.	No cytoplasm stained around nucleus in H&E. Cell body fully visualized via Iba1.	A thin rim of cytoplasm may be stained immediately adjacent to the nucleus.	No cytoplasm stained around nucleus.

### i. Microglia

The microglial cells are readily distinguished from other cellular populations based on the presence of Iba1 staining. On H & E stained sections, microglial nuclei range from 2 µm to 6 µm in diameter, which is typically larger than oligodendrocytes and smaller than astrocytes (Figure 2, Table 4). The microglial nuclei are moderately heterochromatic, containing approximately 3–6 large, dark granules of chromatin and a moderate density of smaller, lighter granules of chromatin. The H & E staining intensity of the microglial nuclei is also intermediate to that of oligodendrocytes and astrocytes. Microglial nuclear shape is highly variable, with spherical, elliptical, irregularly shaped, and triangular nuclei. Due to the variability in microglial nuclear characteristics, and the fact that they are often intermediate to those of oligodendrocytes and astrocytes, we used Iba1 staining exclusively in our final stereological quantification to identify microglia.

However, in addition to marking resident microglia, Iba1 also marks monocytes that have infiltrated into the brain across the blood-brain barrier [40], at which point they are commonly referred to as macrophages. In the current study, we excluded perivascular (blood vessel-associated) macrophages from our stereological quantification. Perivascular macrophages can be distinguished from microglia via two criteria. First, the perivascular macrophages have a rod-shaped morphology that is aligned with a blood vessel. (The blood vessels are visible because they contain numerous H & E-stained endothelial cell nuclei that are linearly aligned with each other). Second, the perivascular macrophages extend all of their processes close and parallel to the neighboring blood vessel. Normal microglia extend their processes evenly in all directions from the cell body.

We qualitatively examined the microglia in each case for signs of activation (Figure 3) (reviews: [21]–[22]). Resting microglia have small and spherical cell bodies, and the processes are thin and well defined (Figure 3A). Slightly activated microglia have marginally thickened but relatively straight processes, with a few small swollen spots visible (Figure 3B). Moderately activated microglia have noticeably enlarged cell bodies, with thick, unevenly swollen, tortuous processes (Figure 3C–D). Strongly activated microglia have further enlarged cell bodies and a loss of processes (Fig 3E). The remaining processes are short and thick, as the cell approaches an amoeboid morphology. Finally, extremely activated microglia have an even larger cell bodies and few if any processes, appearing to largely assume an amoeboid morphology (Figure 3F).

### ii. Astrocytes

We distinguished astrocytes from the other glial populations based on their nuclear morphology in the H & E counterstain (Table 4). The astrocyte nuclei are typically 6–10 µm in diameter, with a minimum diameter of 5 µm. They have a high density of small, light granules of chromatin, but no more than 4 large distinct, dark clusters of chromatin. They are lightly stained overall by H & E compared to other glial cell nuclei and elliptical or spheroid in shape (Figure 2). We confirmed the reliability of our definitions by examining H & E stained nucleus morphology in an adjacent series of tissue sections stained with GFAP immunoreactivity (Figure 2).

### iii. Oligodendrocytes

Oligodendrocyte nuclei are distinguished from astrocytes and microglia based on their nuclear morphology in the H & E counterstain. Our definition of oligodendrocyte nuclei is consistent with previously published definitions in Nissl-stained tissue [38]–[39], [41] (Table 4). The oligodendrocyte nuclei are typically 2.5 µm–5 µm in diameter, with a maximum diameter of 6 µm. They have a high density of large, dark granules of chromatin (typically 5–15), with a low density of smaller, lighter granules compared to other glial nuclei (Figure 2). They stain more darkly via H & E than any other nucleus type, and have a dark, distinct nuclear boundary and are primarily spherical or oval in shape.

### iv. Endothelial cells

Endothelial cell nuclei are distinguishable from all other glial nuclei primarily by their morphology, which is typically either rod-shaped or folded over, with angular borders (Figure 2B). Occasionally, they may appear ovoid in large blood vessels. Because of their association with blood vessels, each endothelial cell nucleus also typically appears in linear alignment with several others (Figure 2B). The endothelial cell nuclei are typically as large as astrocyte nuclei in at least one axis (>5 µm), and sometimes much larger (up to 25 µm). They contain only small, light granules of chromatin at a low to moderate density, with no large, dark granules visible. They are the most lightly stained of all nucleus types examined. Their nuclear boundaries are distinct and dark relative to their staining intensity.

### Cell Numbers

**Figure 4:**
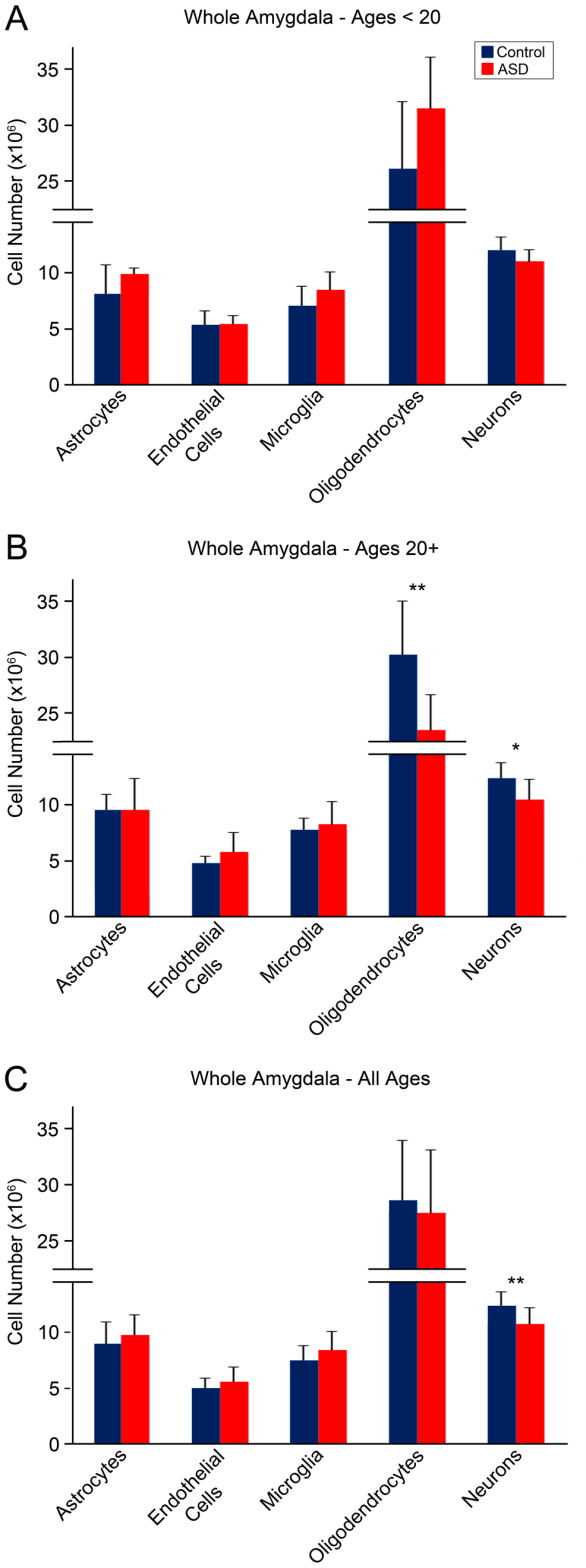
Summary of cell numbers in whole amygdala of ASD and typically developing control brains. A. Adolescents <20 years of age, no significant differences between ASD and controls in any cell population. B. Adults>20 years of age, significantly fewer oligodendrocytes in ASD than typically developing brains (p = .04), trend towards fewer neurons in ASD adults compared to controls (p = .10). C. Adolescents and adults combined, significantly fewer neurons in ASD than control cases (p = .03). Error bars reflect standard deviation. Neuron number data originally published in [17], [35].

Average population numbers for each cell type examined in the whole amygdala and lateral nucleus are displayed in Figure 4. Individual case data for each cell type in the amygdala as a whole is reported in Table 2. Raw average population numbers, nuclear volumes, and somal volumes (when available) for each cell type examined in each amygdala subdivision are reported in Table 5.

**Table 5:** Average cell number data for all cell types and amygdala subdivisions examined.

Diagnosis	Cell Type	Whole Amygdala	Lateral Nucleus	Basal Nucleus	Accessory Basal Nucleus	Central Nucleus	Other Nuclei	Cell Nucleus Volume	Cell Body Volume
Control	Microglia	7.46+/−1.34	2.26+/−0.57	1.83+/−0.40	0.80+/−0.15	0.16+/−0.07	2.30+/−0.42	162+/−30	275+/−45
	Astrocyte	9.00+/−1.96	2.91+/−0.80	2.04+/−0.56	0.92+/−0.19	0.18+/−0.07	2.87+/−0.73	238+/−36	
	Oligodendrocyte	28.6+/−5.43	10.5+/−2.02	6.93+/−1.59	2.58+/−0.58	0.57+/−0.12	7.58+/−1.98	117+/−16	
	Endothelial Cell	4.99+/−0.93	1.69+/−0.44	1.19+/−0.24	0.56+/−0.13	0.11+/−0.03	1.37+/−0.25	259+/−36	
	Neuron	12.21+/−1.28	4.00+/−0.40	3.24+/−0.52	1.28+/−0.20	0.36+/−0.08	3,33+/−0.53		
ASD	Microglia	8.39+/−1.69	2.61+/−0.77	2.02+/− 0.49	0.93+/−0.22	0.20+/−0.08	2.63+/−0.56	150+/−27	275+/−79
	Astrocyte	9.73+/−1.89	3.31+/−0.96	2.25+/−0.32	0.99+/−0.08	0.20+−/0.06	2.98+/−0.89	247+/−40	
	Oligodendrocyte	27.5+/−5.63	10.7+/−2.97	6.26+/−1.34	2.49+/−0.56	0.67+/−0.20	7.42+/−1.53	115+/−17	
	Endothelial Cell	5.60+/−1.28	1.97+/−0.39	1.34+/−0.31	0.60+/−0.17	0.14+/−0.06	1.55+/−0.50	261+/−37	
	Neuron	10.5+/−1.46	3.36+/−0.56	2,84+/−0.58	1.07+/−0.26	0.34+/−0.05	2.92+/−0.32		

All number values are in millions (x10^6^). All volume values are µm^3^. +/− values are the standard deviation. Neuron number data was originally published in [17], [35]. Equivalent density values are presented in Table S2.

### i. Microglia

There were 7.5×10^6^ microglia on average in the typically developing control cases, compared with 8.4×10^6^ microglia on average in the ASD cases (not significant, ns) (Figure 4, Table 5). In the lateral nucleus specifically, there were 2.26×10^6^ microglia on average in typically developing control cases, compared with 2.61×10^6^ microglia in ASD cases (ns) (Table 5).

Microglial somal volumes were highly heterogeneous in all cases examined. Both the typically developing control and ASD cases had an average microglial cell body volume of 275 µm^3^ (ns) (Table 5).

**Figure 5:**
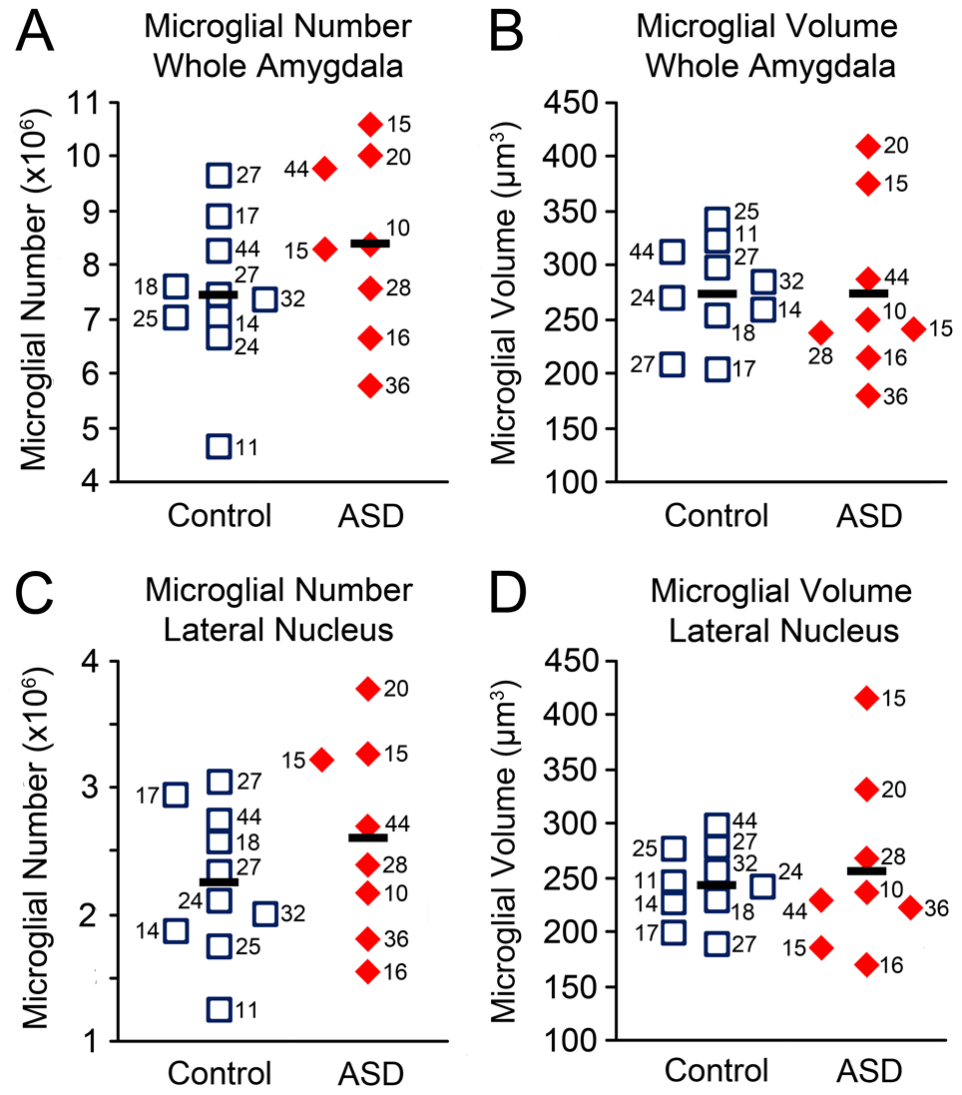
Microglial number and size stereological measures in ASD and typically developing amygdala. Whole amygdala: A. microglial number and B. average microglial somal volume. Two ASD cases with the highest microglial numbers also have the highest microglial volumes. Lateral nucleus: C. microglial number and D. average microglial somal volume. Numbers next to case markers indicate subject age.

**Figure 6:**
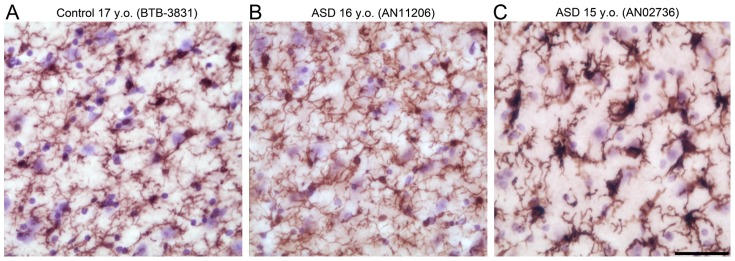
Low magnification fields of view comparing Iba1-positive microglial morphologies. A. Typically developing amygdala (BTB-3831, 17 y.o.) with minimal microglial activation. B. ASD amygdala (AN11206, 16 y.o.) with minimal microglial activation. C. ASD amygdala with strong microglial activation (AN02736, 15 y.o.). Scale bar: 50 µm.

Two ASD cases, AN00764 (20 y.o.) and AN02736 (15 y.o.), had average microglial somal volumes that were above the high end of the typically developing control range (by 19.9% and 10.1%, respectively) (Figure 5, Table 2). Examining these cases qualitatively, many of the microglial cells had a strongly activated morphology (Figure 6). These two cases also had the highest microglial numbers of any cases examined, which were ∼4.2% and ∼10.1% above the high end of the control range (Figure 5, Table 2). A third ASD case, AN06746, had a microglial number that was 1.6% above the high end of the control range (Figure 5A, Table 2), but an average microglial volume in the middle of the control range. AN02736 and AN00764 were also outliers for both microglial volume and number in the lateral nucleus specifically (Figure 5C–D).

### ii. Oligodendrocytes

There were 28.6×10^6^ oligodendrocytes on average in the amygdala of typically developing control cases, and 27.5×10^6^ in ASD cases (ns) (Figure 4, Table 5). Examining lateral nucleus specifically, there were 10.5×10^6^ oligodendrocytes on average in control cases, compared with 10.7×10^6^ oligodendrocytes on average in ASD cases (ns) (Table 5). The average oligodendrocyte nucleus volume was 117 µm^3^ in control cases, and 115 µm^3^ in ASD cases (p = .90) (Table 5).

### iii. Astrocytes

There were 9.00×10^6^ astrocytes on average in the amygdala of control cases, and 9.73×10^6^ astrocytes on average in ASD cases (ns) (Figure 4, Table 5). Examining lateral nucleus specifically, there were 2.91×10^6^ astrocytes on average in control cases, and 3.31×10^6^ astrocytes on average in ASD cases (ns) (Table 5). The average astrocyte nucleus volume was 238 µm^3^ in control cases and 247 µm^3^ in ASD cases (ns) (Table 5).

### iv. Endothelial Cells

There were 4.99×10^6^ endothelial cells on average in the amygdala of typically developing control cases, and 5.60×10^6^ endothelial cells on average in the amygdala of ASD cases (ns) (Figure 4, Table 5). Examining the lateral nucleus specifically, there were 1.69×10^6^ endothelial cells on average in control cases and 1.97×10^6^ endothelial cells on average in ASD cases (ns) (Table 5). The average endothelial cell nucleus volume was 259 µm^3^ in control cases and 261 µm^3^ in ASD cases (ns) (Table 5).

### v. Potential Confounds and Covariates

We examined postmortem pre-fixation interval (PMI) as a possible confound for the cell number and somal volume findings. PMI did not differ significantly between the ASD and typically developing control groups, nor was it significantly correlated with any outcome measure. We did not fully examine hemisphere effects because there was only one control case that had tissue drawn from the right hemisphere. However, within the ASD group, the source hemisphere did not covary significantly with any outcome measure. There was insufficient information on two cases regarding possible seizure history (AN02736 and CAL101; see Information S1). However, none of the ASD cases had a diagnosis of epilepsy, nor was it the primary cause of death. We were not able to examine quantitative relationships with clinical characteristics or outcomes, genetic profile, immunological features, brain mass, or postmortem tissue pH due to insufficient data. We did not find any clear qualitative relationships with clinical characteristics or outcomes (Information S1).

### Correlations

**Figure 7:**
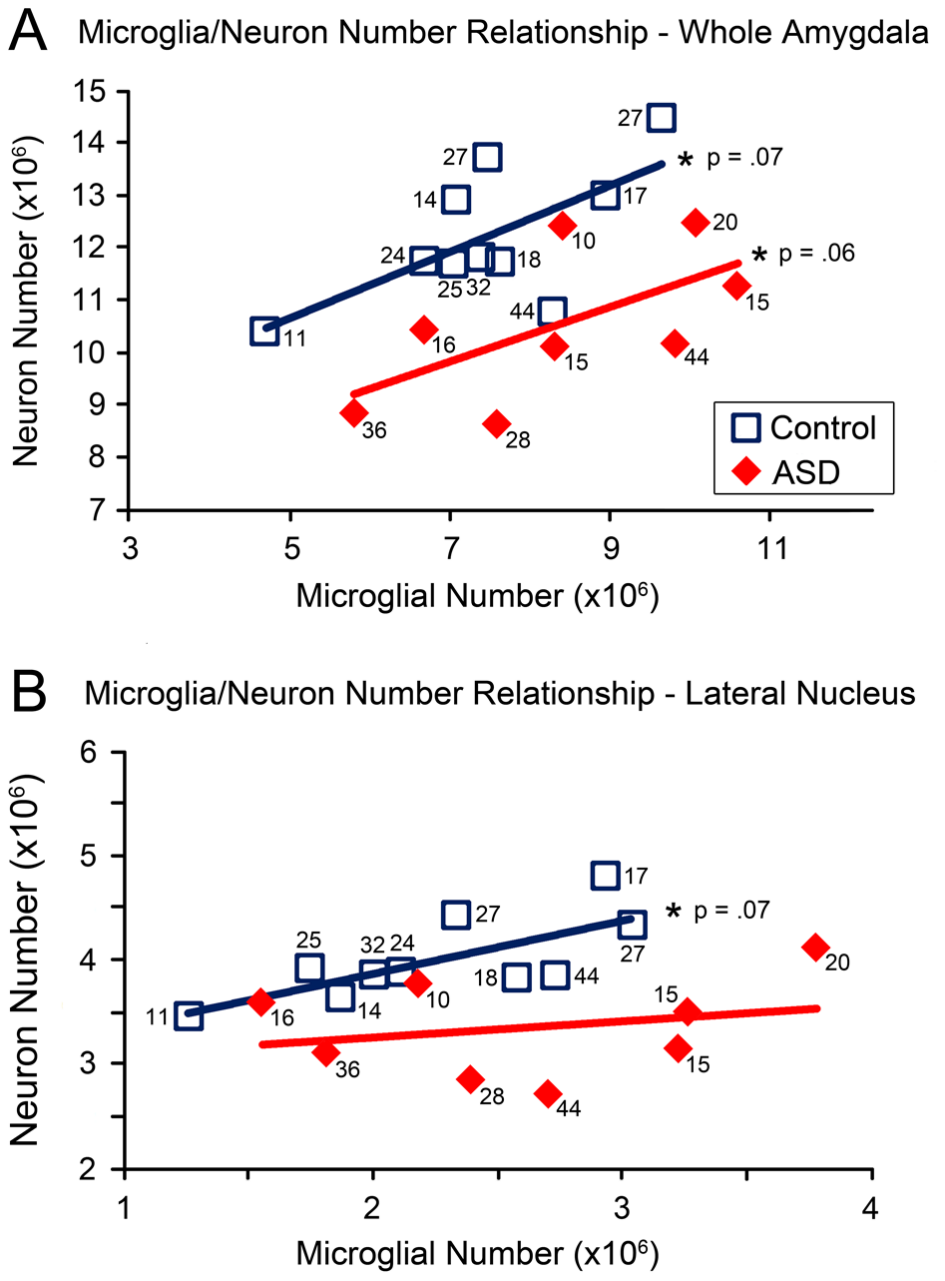
Relationship between microglial number and neuron number in ASD and typically developing cases. A. Trend positive correlation in microglial and neuron number in both typically developing control (p = .06) and ASD (p = .07) whole amygdala and B. in typically developing (p = .07) but not ASD lateral nucleus. Numbers next to case marker indicate subject age.

There were positive trend correlations between neuron number and microglial number in both typically developing control cases (r(10) = .61; p = .06) and ASD cases (r(8) = .67; p = .07) (Figure 7A). There was also a positive trend correlation between microglial number and neuron number in the lateral nucleus of typically developing cases only (r(10) = 0.60, p = .07) (Figure 7B). In the ASD cases, the correlations between neuron number and microglial number were significantly positive in the accessory basal nucleus (r(8) = 0.86, p = .01), basal nucleus (r(8) = 0.91, p<0.01), and the other nuclei grouping (r(8) = 0.91, p<0.01).

### Age-related Analyses

**Figure 8:**
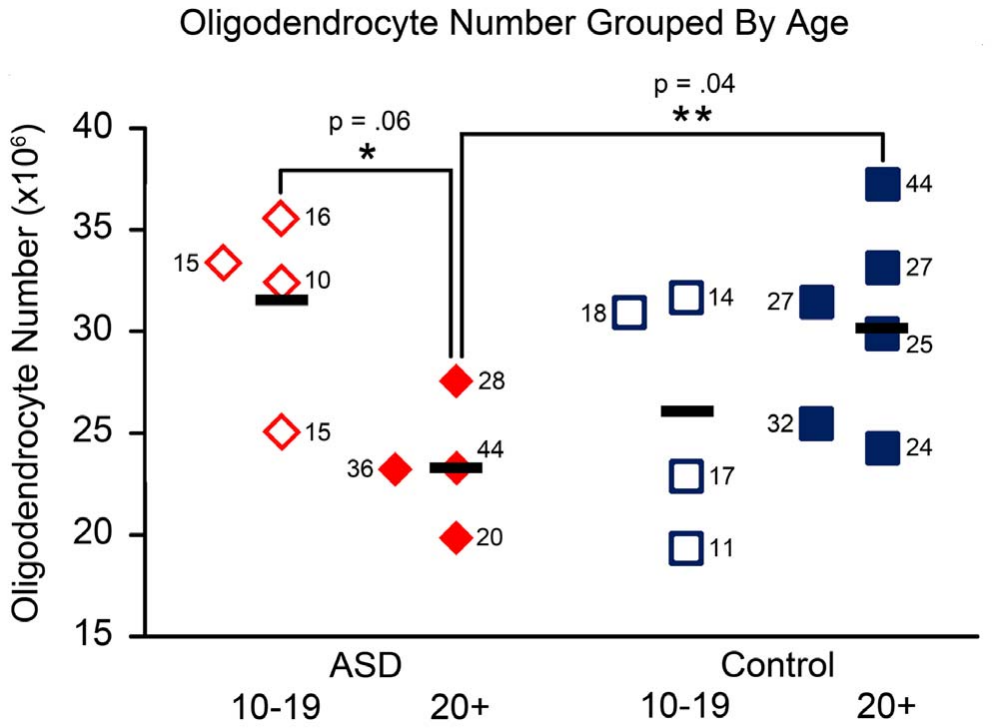
Oligodendrocyte number in the whole amygdala of ASD and typically developing control cases, grouped by age. Oligodendrocyte number is significantly lower in the adult ASD group ages 20+ compared to typically developing controls ages 20+ (p = .04). There is a trend towards lower oligodendrocyte numbers in the adult ASD group compared to the adolescent ASD group ages 10–19 (p = .06). Numbers next to case markers indicate subject age.

There were fewer oligodendrocytes in adult ASD cases older than 20 years than adult typically developing control cases (p = .04) (Figure 8). This effect was significant in the basal nucleus (p = .04) specifically, with a trend in the lateral nucleus (p = .07). There was a trend towards fewer oligodendrocytes in the adult ASD subgroup compared to the adolescent ASD subgroup ages 10–19 (p = .06) (Figure 8). No age-related effects were observed in microglia, astrocytes, or endothelial cells.

## Discussion

In this study, we examined the microglial, oligodendrocyte, astrocyte, and endothelial cell populations in the amygdala of postmortem brains from adolescent and adult male individuals that either had ASD or were typically developing controls. There were three primary objectives in this study: 1) to provide detailed anatomical definitions and stereological measurements for each cell population, 2) to determine if there were differences between typically developing control brains and the ASD diagnostic group in cell size and number, 3) to examine if non-neuronal cell measures were correlated with previously reported neuron number estimates, and 4) to investigate age-related differences in these populations in either typically developing control or ASD brains.

This study is, to our knowledge, the first comprehensive stereological examination of the major glial populations to use a neuroanatomical protocol that distinguishes the microglial population via Iba1 staining [37]–[39]. Because microglial nucleus morphologies overlap with those of the astrocyte and to some extent oligodendrocyte populations [38], this approach substantially improves confidence in the identification of all three major glial types. The protocol is a reliable method for segregating the major glial cell populations that may be applied to future stereological studies of many other brain regions and cohorts. This is also the first study to stereologically examine the numbers of individual glial populations in the typically developing amygdala, rather than reporting densities, which may be susceptible to variable tissue shrinkage [38]. We found that there are on average 7.5 million microglia, 28.6 million oligodendrocytes, 9.0 million astrocytes, and 5.0 million endothelial cells in the amygdala of typically developing adult, male control subjects 11–44 years of age. This is compared to 12.2 million neurons in our prior paper [35]. These estimates may serve as a baseline for future studies of glia in neurodevelopmental and psychiatric disorders, such as schizophrenia and major depressive disorder, where neuron number and oligodendrocyte density abnormalities have been reported in the amygdala [38], [42]–[44]. Similarly, comparative studies of amygdala glia numbers in animal models (e.g., [41], [45]–[47]) or typical human development may use these numbers as a point of comparison.

For our study of adolescent and adult ASD brains, there are no group differences with typically developing control brains in any of the cell populations we examined. There are, however, three primary findings in this study: 1) two ASD brains display evidence of aberrant microglial morphology and number, 2) microglia number demonstrates a trend positive correlation with neuron number in both ASD and control cases, and 3) oligodendrocyte numbers are reduced in adults with ASD relative to typically developing adults. The microglial activation and oligodendrocyte number findings should be considered preliminary due to the small subgroup sizes involved. Below we discuss our findings in each of the cell populations examined. The findings emphasize the need for future comprehensive studies of the amygdala in ASD over the course of development in larger cohorts.

### Microglia

Two out of the eight ASD brains examined, AN00764 (20 y.o.) and AN02736 (15 y.o.), exhibit strong microglial activation with both larger and more numerous microglia than the maximum typically developing control values. This activation is also unlikely to be an artifact of postmortem changes specific to these cases. First, there is no correlation between postmortem interval and either microglial number or average volume in either ASD or typically developing control brains. Second, there are no signs of excessive activation in any typically developing control brains even though many of them had a postmortem pre-fixation interval longer than 24 hours. Third, the ASD brain with the longest postmortem interval, near 48 hours (AN11206), demonstrates no alteration in either microglial number or somal size. By contrast, a brain with a postmortem interval of only 3 hours (AN02736) demonstrates substantial microglial activation.

Although preliminary, our findings, in combination with previous studies of microglia in the disorder, suggest that excessive microglial activation may be present in a subset of patients. Approximately four out of thirteen cases appeared to be similarly strongly affected in a prior study of frontal cortex [18]. This finding is also consistent with observations of global heterogeneity in immune abnormalities in ASD (reviews: [48]–[50]). However, a recent study of the frontoinsular and visual cortices in ASD found a much higher frequency of aberrant microglial activation, with the large majority of cases consistently affected [19]. What might account for this discrepancy? It is possible that activation may be more likely to be present in some brain regions than others in ASD. However, since microglial activation appears to be present in only a subset of ASD cases, the difference may also reflect the particular collections of cases examined in these papers. Therefore, at present we cannot be certain what underlies the discrepancy. Additional studies of microglial features in an expanded number of ASD brains are needed to determine precisely how frequently microglial activation is present in the disorder, and whether it is consistently present across many regions of the brain.

This question is further complicated by the fact that the two ASD cases that show strong signs of excessive microglial activation in this study may each have a different profile of activation. One of the ASD cases (AN02736, 15 y.o.) that appeared to have strong activation had the highest microglial number but second highest average volume, while the other (AN00764, 20 y.o.) had fewer microglia but a higher average somal volume. A third ASD case (AN07646, 44 y.o.) had a microglial number just above the high end of the typically developing control range, but an average microglial somal volume in the middle of the range. Increases in microglial number typically occur over long periods of chronic activation, while profound changes in microglial morphology can happen within a few days (reviews: [15], [46]). Therefore, these cases might reflect different profiles of activation. AN02736, with a higher microglial number but lower average microglial somal volume, might have more chronic but less intense activation. AN00764, with higher average microglial somal volume and lower microglial number, might have more severe but recent activation. Alternately, the degree of microglial activation could vary over time in ASD. If this were true, the cases might have been in slightly different phases of activation when they passed away. Unfortunately, due to the small number of activated cases examined in this study, it's unclear which of these scenarios is correct. Additional studies of microglial features in a large number of ASD brains are needed to determine whether there is heterogeneity in microglial activation profiles in the disorder. Gene expression, cytokine, and PET studies of microglial activation in ASD [20], [23]–[25] can also help to elucidate whether there are distinct profiles of microglial activation.

### Microglia/Neuron Relationships

What roles might excessive microglial activation be playing in the ASD patients where it is present? We initially predicted that excessive microglial activation in the ASD amygdala might be related to our previous finding of lower neuron numbers in the same cohort of cases [17]. This was based on multiple studies of several neurodegenerative disorders describing both excessive microglial activation and neuron loss [26]–[28]. In addition, microglia and neurons are more closely spatially associated in ASD frontal cortex than in typical brains [8]. Therefore, we expected that the activated microglia might be performing phagocytic functions on damaged or dying neurons. In fact, contrary to our hypothesis, increased microglial number is not negatively correlated with neuron number, but instead has a trend positive correlation in both ASD and typically developing brains.

If microglial activation is not associated with lower neuron number in ASD, what might the close spatial relationship of these two populations in the disorder reflect? Activated microglia may naturally move closer to neurons, while not significantly affecting their survival or activity. In this case, microglial activation might reflect activation of the broader immune system in some ASD patients. This could be due to any of several causes: increased sensitivity to pro-inflammatory factors, a pro-inflammatory infection of the brain, a disruption of the blood-brain barrier, or a reaction downstream from the development of autoimmunity to one or more brain proteins (e.g., [51]–[55]). Alternately, activated microglia might be playing a protective role. Microglia can protect neurons with synaptic abnormalities [56] by regulating their function via release of trophic factors and cytokines [57]–[58] or stripping excess synapses [59]–[61].

### Oligodendrocytes

We found that there are a lower number of oligodendrocytes in the amygdala of adults with ASD relative to age-matched typically developing controls. There is also a trend towards lower numbers in adult ASD relative to adolescent ASD cases. These findings must be considered preliminary due to the small subgroup sizes, particularly given that there are no differences in oligodendrocyte number when the age groups are combined. Nevertheless, it will be important to determine in future studies whether there may be a decline in oligodendrocyte numbers in older individuals with ASD. A loss of oligodendrocytes could significantly impact the functional connectivity of the amygdala and present significant cognitive difficulties for older patients with ASD [62]–[63]. If present, this alteration could be due to environmental perturbations, such as elevation of pro-activation cytokines like TNF-α, to which oligodendrocytes are highly vulnerable ([32], although this finding requires further investigation [64]). Additional studies examining the oligodendrocyte population across the lifespan in ASD are needed in order to determine whether there are abnormalities in this population in older patients with the disorder.

### Astrocytes and Endothelial Cells

The absence of significant alterations in the astrocyte and endothelial cell populations in ASD is also noteworthy. The absence of group differences in the astrocyte population does not necessarily indicate that there is no astrocyte activation in ASD, as astrocytes do not proliferate significantly when they are activated. Prior studies have found both qualitative morphology changes that suggest activation and an increase in the levels of GFAP, a protein whose expression increases during astrocyte activation, in ASD [20], [30]. The absence of a difference in endothelial cell numbers, meanwhile, suggests that there is no grossly abnormal vascularization in the amygdala in ASD.

### Summary

In this comprehensive study of non-neuronal cells in the amygdala of adolescents and adults with ASD, we found no global differences in cell numbers and sizes. However, we found alterations in both the microglia and oligodendrocyte populations in subsets of ASD patients that require further investigation. It is now critical to describe the full developmental trajectory of glial and neuronal abnormalities in ASD across multiple brain regions. In particular, which cellular abnormalities are present in pediatric cases, at an age when the behavioral characteristics of the disorder first manifest? However, our findings make it clear that it is important to study cohorts of older people with ASD as well. Alterations such as a reduction in oligodendrocyte numbers later in life might reflect subtle but increasing cognitive difficulties that have not yet been described. Given consistent evidence of heterogeneity in microglial activation, future studies must increase the number of brains available in order to dissect out possible phenotypic subgroups in the ASD population and investigate behavioral, immunological, and genetic correlations. This knowledge will provide substantial insight into the underlying neurobiology of ASD, pointing the way towards effective phenotype identification leading to targeted pharmacological intervention.

## Supporting Information

Information S1 **Capsule clinical summaries of ASD cases.** All information is drawn from the Autism Tissue Portal (http://www.atpportal.org).
(DOCX)

Table S1 **Cell density data for the whole amygdala in individual cases, sorted by diagnosis and age.** All density values are cells per mm^3^.
(DOCX)

Table S2 **Average cell density data for all cell types and amygdala subdivisions examined.** All density values are cells per mm^3^. +/− values are the standard deviation.
(DOCX)

Data S1 **Raw cell number, nucleator, nuclei volume, and covariate data for all cases.**
(XLSX)
