# Supplementary material for: Stereological Study of Amygdala Glial Populations in Adolescents and Adults with Autism Spectrum Disorder
Source: PLoS One. 2014 Oct 17;9(10):e110356. doi: 10.1371/journal.pone.0110356 (PMC4201518; doi:10.1371/journal.pone.0110356)
Supplement: Information S1 — Capsule clinical summaries of ASD cases. All information is drawn from the Autism Tissue Portal (http://www.atpportal.org). (DOCX) [file pone.0110356.s001.docx]

BTB-3714 - DSM-IV diagnosis, age 10 at death. Language marked by echolalia. Unusual preoccupations. Diagnosed as mentally retarded at 9 years of age. Delayed motor skills. Behavior marked by hyperactivity, tantrums, and impulse control difficulties at 3 years 10 months of age. No information available regarding regression, comorbidities, or family history.

AN02736 - ADI-R diagnosis, age 15 at death. Nonverbal, learned 3 words during lifetime. Several communicative gestures learned. Extreme interests, strong sensory aversions, frequent self-stimulating behaviors. Severe motor delays, did not walk until age 7. No regression reported. Behavior marked by occasional self-injurious hand-biting. Parentally reported grand mal seizures every 3-4 years from age 2 that were unrelated to the cause of death. Frequent ear infections, difficulty eating.

UCD H499 - ADI-R diagnosis, age 15 at death. No additional information available.

AN11206 - ADI-R diagnosis, age 16 at death. 4-5 words spoken regularly as an adult, no echolalia, 40-50 word receptive language repertoire and ability to answer by writing single words. Limited repetitive behaviors. Normal motor development. Regression reported at 18 months; language development had previously been normal. Behavior marked by occasional self-injurious hand biting when younger. One aggression incident. Obese. Five typically developing siblings.

AN00764 - ADI-R diagnosis, age 20 at death. Delayed language following regression, with limited repertoire of several dozen single words not used on daily basis; learned gestures to make requests as an adult. Repetitive, ritualistic, and self-stimulatory behaviors, as well as unusual sensory interests. Low functioning cognitively. Normal early motor development followed by later motor delays, particularly in fine motor skills. Regression reported at 12 months. Some self-injurious picking and hand-biting behavior. Erratic sleeping patterns. Two typically developing siblings.

UMB-4226 - ADI-R diagnosis, age 28 at death. Normal language development. Some unusual preoccupations. High functioning, enrolled in community college after finishing high school but did not complete it. Normal motor development. No regression reported. No comorbidities reported. Substance abuse in early adulthood. Three typically developing siblings.

CAL101 - ADI-R diagnosis, age 35 at death. Language abilities included single words but no phrase speech; had some gesture abilities and ability to use picture selection to communicate. Multiple special interests. Sensitive to sound. Low functioning, development at the level of a 42 month old at 9 years of age. No motor delays. No regression reported. Co-diagnosed with obsessive-compulsive disorder. Parentally reported occasional seizures from 18-28 years of age, at which point seizure medication was discontinued with no additional seizures. Socially avoidant. Younger sister with ASD, possible maternal rubella during pregnancy.

AN16961 - ADI-R diagnosis, age 36 at death. No language, but learned some signs and gestures to make requests as an adult. Multiple repetitive and self-stimulating behaviors. Low functioning, diagnosed with severe mental retardation. No motor delays during early development. Regression reported at 16 months after achieving first words and toilet training. Multiple severely self-injurious behaviors. Suffered from hereditary kidney failure. Two typically developing siblings.

AN06746 - ADI-R diagnosis, age 44 at death. Delayed language development with pronounced echolalia, but spoke full sentences and was able to follow multistep commands as an adult. Narrow range of interests, strong preoccupations, and frequent sensory repetition and self-stimulation during childhood. Tested IQ of 66. Moderate delays in motor milestones. No regression reported. Obese. Behavior described as talkative, excitable. Four typically developing siblings.
