# Supplementary material for: Stereological Study of Amygdala Glial Populations in Adolescents and Adults with Autism Spectrum Disorder
Source: PLoS One. 2014 Oct 17;9(10):e110356. doi: 10.1371/journal.pone.0110356 (PMC4201518; doi:10.1371/journal.pone.0110356)
Supplement: Table S1 — Cell density data for the whole amygdala in individual cases, sorted by diagnosis and age. All density values are cells per mm3. (DOCX) [file pone.0110356.s002.docx]

| **Case** | **Diagnosis** | **Age** | **Microglia Density** | **Oligodendrocyte Density** | **Astrocyte Density** | **Endothelial Cell Density** | **Neuron Density** |
| --- | --- | --- | --- | --- | --- | --- | --- |
| UCD H204 | Control | 11 | 3814 | 15771 | 5887 | 31223 | 8484 |
| BTB-3830 | Control | 14 | 4214 | 18830 | 2918 | 28423 | 8392 |
| BTB-3831 | Control | 17 | 6170 | 15766 | 7193 | 32968 | 8984 |
| BTB-3851 | Control | 18 | 5494 | 22285 | 7320 | 38459 | 8485 |
| BTB-3809 | Control | 24 | 4559 | 16547 | 4994 | 29408 | 8571 |
| BTB-4016 | Control | 25 | 5363 | 22776 | 7741 | 39309 | 8923 |
| BTB-3849 | Control | 27 | 5980 | 20514 | 6600 | 36235 | 8983 |
| BTB-3706 | Control | 27 | 5059 | 21377 | 6054 | 35049 | 9314 |
| BTB-3966 | Control | 32 | 5554 | 19208 | 8427 | 36799 | 8918 |
| UCD H1901 | Control | 44 | 6044 | 27201 | 6724 | 44131 | 7861 |
|  |  |  |  |  |  |  |  |
| **Mean** | Control | 23.9 | 5225 | 20028 | 6386 | 3562 | 8692 |
|  |  |  |  |  |  |  |  |
| BTB-3714 | ASD | 10 | 5312 | 20544 | 6083 | 4079 | 7867 |
| AN02736 | ASD | 15 | 10151 | 23753 | 9820 | 4531 | 10823 |
| UCD H499 | ASD | 15 | 5190 | 20817 | 5764 | 3038 | 6319 |
| AN11206 | ASD | 16 | 4511 | 24039 | 7096 | 3776 | 7057 |
| AN00764 | ASD | 20 | 7145 | 14130 | 6820 | 4616 | 8856 |
| UMB-4226 | ASD | 28 | 5646 | 20579 | 5870 | 3505 | 6438 |
| CAL101 | ASD | 35 | N/A | N/A | N/A | N/A | 8328 |
| AN16961 | ASD | 36 | 5694 | 22956 | 7178 | 4022 | 8725 |
| AN06746 | ASD | 44 | 6055 | 14370 | 8374 | 4931 | 6301 |
|  |  |  |  |  |  |  |  |
| **Mean** | ASD | 23 | 6213 | 20149 | 7126 | 4062 | 7857 |
