# Supplementary material for: Stereological Study of Amygdala Glial Populations in Adolescents and Adults with Autism Spectrum Disorder
Source: PLoS One. 2014 Oct 17;9(10):e110356. doi: 10.1371/journal.pone.0110356 (PMC4201518; doi:10.1371/journal.pone.0110356)
Supplement: Table S2 — Average cell density data for all cell types and amygdala subdivisions examined. All density values are cells per mm3. +/− values are the standard deviation. (DOCX) [file pone.0110356.s003.docx]

| **Diagnosis** | **Cell Type** | **Whole Amygdala** | **Lateral Nucleus** | **Basal Nucleus** | **Accessory Basal Nucleus** | **Central Nucleus** | **Other Nuclei** |
| --- | --- | --- | --- | --- | --- | --- | --- |
| Control | Microglia | 5225 +/-  804 | 4885 +/-  1014 | 5260 +/-  961 | 5170 +/-  754 | 4608 +/-  1394 | 5661 +/-  772 |
|  | Astrocyte | 6386 +/-  1564 | 6407 +/-  1967 | 5884 +/-  1665 | 6002 +/- 1321 | 5000 +/-  1844 | 7052 +/-  1501 |
|  | Oligodendrocyte | 20028 +/-  3600 | 22840 +/-  4502 | 19830 +/-  3969 | 16688 +/-  3233 | 16466 +/-  2456 | 18527 +/-  3865 |
|  | Endothelial Cell | 3562 +/-  928 | 3698 +/-  1023 | 3466 +/-  864 | 3698 +/-  1040 | 3356 +/-  1121 | 3471 +/-  1000 |
|  | Neuron | 8692 +/-  414 | 8716 +/-  821 | 9244 +/-  880 | 8254 +/-  792 | 10253 +/-  1492 | 8220 +/-  788 |
|  |  |  |  |  |  |  |  |
| ASD | Microglia | 6213 +/-  1763 | 5880 +/-  1959 | 6083 +/-  1763 | 6196 +/-  1705 | 5901 +/-  2921 | 6683 +/-  1724 |
|  | Astrocyte | 7126 +/-  1385 | 7478 +/-  2442 | 6807 +/-  1391 | 6682 +/-  1384 | 5838 +/-  1762 | 7359 +/-  1266 |
|  | Oligodendrocyte | 20149 +/-  3899 | 23657 +/-  5516 | 18832 +/-  3985 | 16646 +/-  3522 | 19575 +/-  5737 | 18582 +/-  2881 |
|  | Endothelial Cell | 4062 +/-  624 | 4389 +/-  777 | 3968 +/-  521 | 3896 +/-  738 | 3993 +/-  1105 | 3848 +/-  900 |
|  | Neuron | 7857 +/-  1509 | 7642 +/-  1403 | 8578 +/-  1496 | 7815 +/-  1670 | 10368 +/-  2336 | 7548 +/-  1899 |
